# Non‐invasive temporal interference stimulation of the hippocampus in early Alzheimer's disease

**DOI:** 10.1002/alz70859_101007

**Published:** 2025-12-25

**Authors:** Julia Borella, Ketevan Alania, Kaarin Šabad, Ines Violante, Edward Rhodes, Christopher R. Butler, Nir Grossman

**Affiliations:** ^1^ Imperial College London, London United Kingdom; ^2^ University of Surrey, Guildford United Kingdom

## Abstract

**Background:**

Temporal interference (TI) stimulation is a novel method of electrical brain stimulation that is capable of non‐invasively and selectively targeting deep brain structures. Previously, we demonstrated that TI stimulation could modulate hippocampal activity and improve performance on an episodic memory task in healthy young adults. In this study, we investigated the effects of multi‐session TI stimulation of the hippocampus in patients with early‐stage Alzheimer's disease (AD).

**Method:**

Twenty‐one AD patients received 50 minutes of TI stimulation to the left hippocampus daily, over 10 consecutive days. The effects of the intervention were assessed using a battery of neurocognitive assessments and neuroimaging measures, including positron emission tomography (PET) of mitochondrial complex‐1, blood‐oxygen level‐dependent functional magnetic resonance imaging (BOLD fMRI), arterial spin labelling (ASL) and diffusion tensor imaging (DTI).

**Result:**

We demonstrate that multi‐session TI stimulation of the hippocampus is safe and well‐tolerated. Following TI stimulation, AD patients showed selective improvements in hippocampal‐dependent episodic memory tasks.

**Conclusion:**

We report the main outcomes in terms of safety, neurocognitive assessments and neuroimaging measures from the first study of TI stimulation in patients with early‐stage AD.

